# Supplementary material for: Genome-wide CRISPR Screens in T Helper Cells Reveal Pervasive Crosstalk between Activation and Differentiation
Source: Cell. 2019 Feb 7;176(4):882–896.e18. doi: 10.1016/j.cell.2018.11.044 (PMC6370901; doi:10.1016/j.cell.2018.11.044)
Supplement: Data S2. Processed Data from All the Steps of the Analysis, Related to Figure 1 [file mmc2.zip › supplemental data/motif analysis/Stat6_SRX021632_homer/homerResults/motif17.similar.html]

motif17

## Information for motif17

C
T
A
G
G
T
A
C
C
T
A
G
A
G
T
C
G
T
C
A
A
C
G
T
A
C
T
G
A
G
T
C
C
A
T
G
A
G
T
C
T
G
C
A
C
T
G
A
  
Reverse Opposite:  

G
A
C
T
A
C
G
T
C
T
A
G
G
T
A
C
C
T
A
G
A
G
T
C
T
G
C
A
A
C
G
T
A
C
T
G
A
G
T
C
A
C
T
G
A
G
T
C
  

|  |  |
| --- | --- |
| p-value: | 1e-85 |
| log p-value: | -1.979e+02 |
| Information Content per bp: | 1.858 |
| Number of Target Sequences with motif | 1523.0 |
| Percentage of Target Sequences with motif | 6.02% |
| Number of Background Sequences with motif | 849.3 |
| Percentage of Background Sequences with motif | 3.52% |
| Average Position of motif in Targets | 99.8 +/- 53.9bp |
| Average Position of motif in Background | 94.8 +/- 72.3bp |
| Strand Bias (log2 ratio + to - strand density) | -0.0 |
| Multiplicity (# of sites on avg that occur together) | 1.22 |
| Motif File: | file (matrix) reverse opposite |

### Similar de novo motifs found

|  |  |  |  |  |  |  |  |
| --- | --- | --- | --- | --- | --- | --- | --- |
| Rank | Match Score | Redundant Motif | P-value | log P-value | % of Targets | % of Background | Motif file |
| 1 | 0.871 | T G A C C T A G A T G C G T C A C A G T A T C G A G T C A C T G A G T C C T G A | 1e-85 | -197.046183 | 3.83% | 1.91% | motif file (matrix) |
| 2 | 0.851 | A C T G A G T C A C T G G T A C C G T A A C G T A C T G A G T C | 1e-62 | -144.950715 | 5.51% | 3.43% | motif file (matrix) |
